# Monitoring the Damage State of Fiber Reinforced Composites Using an FBG Network for Failure Prediction

**DOI:** 10.3390/ma10010032

**Published:** 2017-01-03

**Authors:** Esat Selim Kocaman, Erdem Akay, Cagatay Yilmaz, Halit Suleyman Turkmen, Ibrahim Burc Misirlioglu, Afzal Suleman, Mehmet Yildiz

**Affiliations:** 1 Faculty of Engineering and Natural Sciences, Sabanci University, Tuzla, 34956 Istanbul, Turkey; esatselim@sabanciuniv.edu (E.S.K.); cagatayyilmaz@sabanciuniv.edu (C.Y.); burc@sabanciuniv.edu (I.B.M.); 2 Faculty of Aeronautics and Astronautics, Ayazaga Campus, Istanbul Technical University, Maslak, 34469 Istanbul, Turkey; erdemakay@itu.edu.tr (E.A.); halit@itu.edu.tr (H.S.T.); 3 Integrated Manufacturing Technologies Research and Application Center, Sabanci University, Tuzla, 34956 Istanbul, Turkey; 4 Sabanci University-Kordsa Global, Composite Technologies Center of Excellence, Istanbul Technology Development Zone, Sanayi Mah. Teknopark Blvd. No: 1/1B, Pendik, 34906 Istanbul, Turkey; 5 Mechanical Engineering Department, University of Victoria, Victoria, BC V8W 2Y2, Canada; suleman@uvic.ca

**Keywords:** polymer-matrix composites, fatigue, mechanical testing, damage monitoring, fibre Bragg grating

## Abstract

A structural health monitoring (SHM) study of biaxial glass fibre-reinforced epoxy matrix composites under a constant, high strain uniaxial fatigue loading is performed using fibre Bragg grating (FBG) optical sensors embedded in composites at various locations to monitor the evolution of local strains, thereby understanding the damage mechanisms. Concurrently, the temperature changes of the samples during the fatigue test have also been monitored at the same locations. Close to fracture, significant variations in local temperatures and strains are observed, and it is shown that the variations in temperature and strain can be used to predict imminent fracture. It is noted that the latter information cannot be obtained using external strain gages, which underlines the importance of the tracking of local strains internally.

## 1. Introduction

Among various classes of composite materials, fibre-reinforced polymeric composites are frequently utilized as structural components in a variety of industries ranging from aeronautics and automotive to civil infrastructure owing to their high specific stiffness and strength. Despite the care shown in the design and manufacturing of these structures using high-tech equipment, composites have certain drawbacks that need to be addressed for their reliable usage at prolonged time scales. Unlike conventional isotropic and homogeneous materials, such as metals and alloys, their mechanical behaviour is complex to model due to their heterogeneous internal structure. Hence, continuous monitoring of the strain state of composites under different environmental conditions and mechanical loads, especially cyclic loads, is particularly important given that such loading is the most common cause inflicting catastrophic damage on composite structures during service. Therefore, it is crucial to develop techniques to monitor or “sense” the strain state of the composite during service and use these data in situ to predict the onset of failure [1].

One of the most recent and precise sensing techniques for strain and structural health monitoring (SHM) of structures is to use fibre Bragg grating (FBG) sensors. FBGs are optical sensors that are sensitive to both strain and temperature (via thermal strains). They are fabricated by periodic modulation of the refractive index of a single mode optical fibre core using Ultraviolet (UV) light [2]. Upon exposure to a broad band (near IR) of light sent through the optical fibre, an FBG sensor acts like a mirror reflecting a narrow light signal centred at a particular wavelength, referred to as Bragg wavelength $\lambda_{B}$. The Bragg wavelength is a function of the grating pitch (Λ) (spacing between periodic variation of refractive index) and effective refractive index ($n_{eff}$) of the sensor and can be formulated as $\lambda_{B}=2n_{eff}\Lambda$. Strain and temperature can be measured based on the shift in the wavelength using the following relation $\frac{\Delta \lambda_{B}}{\lambda_{B}}=\left(1-p_{e}\right)\varepsilon +\left(\alpha +\xi \right)\Delta T$, where Δλ is the change in the wavelength, $p_{e}$ is the photo-elastic coefficient and *α* and *ξ* are the thermal expansion and thermo-optic coefficient of fibres, respectively. *ϵ* is the strain, and ΔT represents the temperature change of the sensor [3]. The sensitivity of strain and temperature of a bare FBG is around 1.2 pm/με and 13.7 pm/°C, respectively; however, it is crucial to measure the strain and temperature sensitivity of every embedded FBG sensor to account for the strain transfer between the sensor and the host material, since factors, such as the variation in material properties and manufacturing conditions, may alter the sensitivity [4].

Being small and flexible, FBG sensors can be embedded discretely into composites at locations of interest, thereby allowing for the tracking of local strain distribution and evolution without compromising the structural integrity of the host material [2]. In [5], we demonstrated the reliability of FBG sensors in fatigue experiments where they could outlast the sample life time, providing real-time strain data for almost more than 5.5 million fatigue cycles. FBG sensors possess several other important attributes, such as immunity to the electromagnetic interference, light weight, multiplexing, absolute measurement capability and high corrosion resistance. Moreover, the same set of embedded FBG sensors can be used for process monitoring of composites, such as resin flow [6], cure monitoring [7,8] and the detection of cure-induced residual strains [7,9], thus assuring the high quality of the manufactured components [10,11]. Furthermore, FBG sensors were successfully used to monitor composite structures under real-time operating conditions, as can be seen in [12,13].

There have been several efforts reported in the open literature proposing to utilize FBG sensors to monitor internal strain states of composite structures [14,15,16,17,18,19,20,21,22,23]. For example, Takeda et al. introduced a new method to predict damage patterns and strains of notched composite laminates with embedded FBG sensors utilizing a layer-wise finite element model [24]. Doyle et al. performed in situ processing and condition monitoring to evaluate different fibre optic sensor systems and obtained a good correlation between strains acquired by surface-mounted optical sensors and external strain gages. They also demonstrated the feasibility of these sensor systems to monitor the stiffness degradation in composites due to fatigue [25]. Baere et al. evaluated the performance of embedded and bonded FBG sensors with organic modified ceramic coating through measuring strains in thermoplastic composites under fatigue loading conditions, wherein it was shown that the two strain quantities agree well with each other, indicating the feasibility of the sensing system [26]. Shin et al. used embedded FBG sensors to monitor fatigue damage evolution in graphite/epoxy composite specimens with a central circular hole and demonstrated the potential of such sensors to detect the occurrence and progress of damage [27]. In Takeda’s study, embedded FBG sensors were used to sense local strain distribution due to transverse crack formation by measuring the power of the reflected light from sensors [28]. In another work, both spectrum and wavelength information received from FBG sensors were utilized for the durability tests (drop-weight impact and two periodic fatigue tests) and for the condition monitoring of a composite wing structure [29].

One important problem with the prediction of failure in a composite structure is that, due to their heterogeneous structure, damage can initiate and progress at multiple locations within the loaded section, unlike metals, where the failure is often caused by the propagation of a single microscopic crack that turns into a macroscopic one later on. Matrix cracking, matrix-fibre debonding, delamination, transverse-ply cracking and fibre breakage are some of the well-known damage forms observed in fibre-reinforced composites exposed to fatigue loads. Moreover, various damage modes can occur independently, concurrently or interactively, making time-based estimates of a potential failure very difficult. Since the damage formation is distributed and progressive, the reductions in strength and stiffness can start at the early stages of the fatigue loading [30]. It is now understood that stiffness degradation of fibre-reinforced polymer matrix composites in response to cyclic loads is characterized by three distinct stages. In the first stage comprising the initial 15%–20% of fatigue life, the rapid formation and interconnection of matrix cracking causes a sharp, non-linear decrease in stiffness. The second stage accounts for 15%–20% up to 90% of the fatigue life where there is a gradual and linear decrease in stiffness, which is attributed to the propagation of several cracks, fibre debonding and delamination. The final stage is distinguished by a sharp nonlinear decrease in stiffness due to the plurality of fibre breakages [31]. Note that this sequence is identical to that of homogeneous metals and alloys, but accompanied by rather different damage mechanisms.

Despite the recent interest in SHM, the number of such studies, particularly about the usage of FBG and similar sensor networks to monitor and understand the governing processes of fatigue, is scarce, and the reported studies are limited to low cycle and low strain amplitude fatigue, fatigue monitored with surface bonded sensors and/or the fatigue of thermoplastic materials with embedded FBG sensors. Correlating the local internal strain readings to external gage data under low-cycle fatigue conditions is important in terms of predicting an approaching failure that would allow time for removing the composite from service. It should also be kept in mind that the stability of the internal sensor network is crucial; as they are exposed to repetitive high strain amplitude dynamic loads, the composite endures [32]. With this motivation, we use local strain data provided by FBG sensors embedded in glass-reinforced composites under constant high strain and low-cycle fatigue loading conditions to focus on the distribution and evolution of strain along the composite specimens, allowing thus to demonstrate the onset of an approaching failure. It is noted that the FBG network provides a useful means to identify local strains, which start to deviate significantly as the fatigue progresses, a sign of an approaching failure, as is shown in this study. In addition, when a specimen is subjected to cyclic loading, a portion of the mechanical energy is dissipated as heat (also referred to as autogenous heating) causing a rise in the temperature of the specimen [33,34,35,36,37,38,39,40,41,42]. This was already demonstrated for metallic materials during the fatigue loading to predict the remaining usable life of the material [41,42]. Upon experimental analysis on the possible mechanisms of heat generation in vibrothermography, several reasons were proposed in the literature, including viscoelasticity/material damping, heating due to the plastic deformation at the end of the crack tips that are formed due to the repeated loading and, finally, the friction between the internal surfaces of the cracks [43,44].

In this work, combined with the data gathered from the FBG network inside several composite specimens composed of biaxial glass fibres and epoxy matrix, we present a hypothesis that the heat generated due to rubbing of the surfaces during the fatigue process is dominant and demonstrate that both the local strain data and rise in temperature can be used to monitor the internal damage state of a fibre glass epoxy composite. It is also shown that the FBG network can remain useful and collect data to track local strains all the way until the fracture of the specimen. Practical issues in relation to performing fatigue testing under constant displacement and strains achieved by using a linear variable differential transformer (LVDT) and extensometer, respectively, as control sensors are also addressed.

## 2. Experimental Section

### 2.1. Specimen Preparation and Testing Equipment

**Figure 1:**
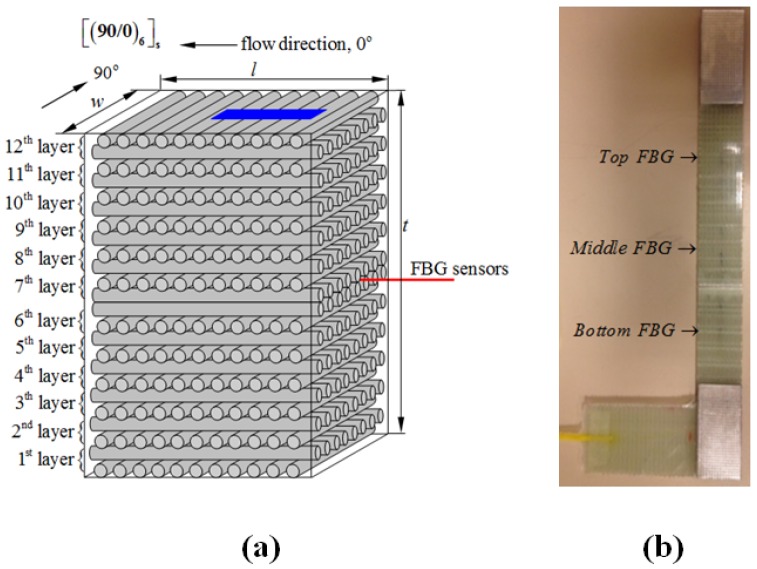
(**a**) The schematic drawing for stacking sequences together with the placement of FBG sensors and also the orientation of the cut test specimen indicated by the blue region where *l*, *w* and *t* indicate the length, width and the thickness of the manufactured composite plate; (**b**) L-shaped specimen that enables easy gripping of the test specimen by the testing system.

In the course of this study, a total of five flat composite panels were manufactured. Four of them were produced using a resin transfer moulding (RTM) method, while the fifth one with a vacuum infusion (VI) technique. Each panel contained two embedded FBG networks (each network includes 3 FBG sensors at different locations), leading to in total 10 test specimens with FBG sensors. Two of the ten specimens were discarded due to FBG sensor failure/breakage at ingress/egress locations. A laboratory-scale RTM apparatus with the capability to embed optical fibres into composite parts was used to produce panels with dimensions of 620 mm × 320 mm × 3.5 mm. Composite laminates consisted of E-glass fibre and epoxy resin and had a stacking sequence of [90/0]_6*S*_. Metyx LT300 E10A 0/90 biaxial E-glass stitched fabric (Metyx Composites, Istanbul, Turkey) was used as the reinforcement, which has an area density of 161 g/m^2^. in the 0° orientation, which is aligned along the resin flow direction in the mould, and 142 g/m^2^ in the 90° orientation, leading to the total area density of 313 g/m^2^. The selected resin system is Araldite LY 564 epoxy resin mixed with XB 3403 hardener (manufactured by Huntsman Corporation, The Woodlands, TX, USA) with the ratio of 100 and 36 parts by weight. The composite panels underwent an initial cure at 65 °C for 24 h with a post cure at 80 °C for 24 h. Three 1 mm-long FBG sensors with Bragg wavelengths of 1540, 1550 and 1560 nm that are written on the same fibre optic cable with 4-cm or 6-cm intervals were purchased from Technica SA (Beijing, China). Prior to manufacturing, the fibre optic cable was fixed onto the 0° surface of a ply through passing it under fibre stitches. The plies were stacked such that the fibre optic cable was between the 6th and 7th layers of the laminate, as shown in Figure 1a. Mechanical test specimens with and without FBG sensors were cut out from the composite panels using a water-cooled diamond circular blade saw into dimensions of 250 mm × 25 mm × 3.7 mm with a 150-mm gage length. The length (also the loading axis) of the specimen was aligned with the 0° fibre orientation. In specimens with FBG sensors, the middle FBG (1550 nm) was positioned at the centre of the gage length of the specimens, and the remaining two sensors were located towards the grips of specimens, as shown in Figure 1b. All three sensors were oriented along the loading direction. To avoid damage and in turn the breakage of test specimens at grip locations, both ends of specimens were tabbed with an aluminium tab having dimensions of 50 mm × 25 mm × 1 mm using a two-component room temperature curing epoxy system (Araldite 2011, Huntsman Corporation, The Woodlands, TX, USA).

All tests were performed on an MTS 322 test frame (MTS Systems Corporation, Eden Prairie, MN, USA) with MTS 647 hydraulic wedge grips using an MTS FlexTest GT digital controller with MTS Station Manager software (FlexTest GT Station Manager Version 3.5C, MTS Systems Corporation, Eden Prairie, MN, USA). Load and displacement data were collected with a built-in load cell (MTS 661.20F-03, MTS Systems Corporation, Eden Prairie, MN, USA) and linear variable differential transformer (LVDT) (MTS Systems Corporation, Eden Prairie, MN, USA), respectively. Strain was collected using an axial extensometer (MTS 634.25F-24, MTS Systems Corporation, Eden Prairie, MN, USA). In some tests, a second axial extensometer (Epsilon 3542, Epsilon Technology Corp., Jackson, WY, USA) was used simultaneously to study the effect of gage length on the strain measurement. The Micron Optics SM130-700 interrogator (Micron Optics, Inc., Atlanta, GA, USA) with Micron Optics Enlight software was used to collect FBG data. K-type thermocouples (OMEGA Engineering, Inc., Norwalk, CT, USA) were used to measure temperature, and corresponding data were collected using a National Instruments NI SCXI-1314 DAQ card (National Instruments Corporation, Austin, TX, USA) in an NI SCXI-1000 chassis with Signal Express software. All data were acquired at a sampling rate of 100 Hz.

### 2.2. Test Procedure

The baseline parameters for fatigue tests were determined by performing eleven static tests whereby the average ultimate tensile stress and strain of the composite specimens were measured to be 320 MPa and 16.31 με, respectively. In this study, eight FBG sensor embedded specimens were extracted intact from five flat composite panels and then were subjected to constant amplitude strain and tension-tension sine wave tests at various strain ratios ($\varepsilon_{max}$/$\varepsilon_{ult}$) varying between 0.5 and 0.6, where the maximum fatigue loading to be imposed on these test specimens was determined based on the strain ratio of interest. To ensure that fatigue tests were performed in tension-tension mode, all specimens were subjected to a minimum stress of 27.6 MPa. Autogenous heating of test specimens becomes a concern during fatigue testing of glass fibre-reinforced composites since the generated heat is not transferred to the environment as fast as in metallic materials. Knowing that fatigue properties of composites are especially sensitive to heat, tests were performed at a frequency of 4 Hz to prevent extensive overheating of samples. Of the eight experiments on composite specimens with embedded FBG sensor networks, three of them were rejected due to experimental errors, such as power outage, unexpected failure and slipping of specimens at grip locations. However, the collected and processed data in these experiments were still useful to observe the fatigue behaviour of specimens at the strain ratio of interest.

For each fatigue specimen, the strain sensitivities of embedded FBG sensors were determined as follows. First, the minimum and maximum loads were calculated using minimum and maximum stress and the area of the specimen. A given specimen was statically tensioned up to the calculated maximum load and then unloaded down to the minimum load while acquiring the displacement data by LVDT, strain data by the extensometer and the Bragg wavelength by the optical interrogator. The loading and unloading procedure is applied for the second time. Then, all of the collected data were processed such that the extensometer data corresponding to the second ramp were plotted as a function of pertinent Bragg wavelength for each FBG and LVDT, and linear regression was used to determine strain calibration coefficients for FBG and LVDT sensors. The strain sensitivities of bottom, middle and top FBGs for specimens L1 , L2, L3, E1 and E2 were determined respectively as (1.24, 1.25, 1.24 in pm/με), (1.27, 1.25, 1.31 in pm/με), (1.25, 1.28, 1.30 in pm/με), (1.27, 1.30, 1.24 in pm/με) and (1.23, 1.26, 1.26 in pm/με), leading to the average of (1.25, 1.27, 1.27 pm/με).

Prior to fatigue testing, the temperature sensitivity of the FBG sensors was determined in order to account for the temperature variation in the specimens due to autogenous heating. To this end, FBG sensors were placed in a furnace. The temperature in the furnace was ramped from 30 °C up to 60 °C and allowed to soak at each 10 °C temperature increment for one hour before the temperature and wavelength were recorded. A plot of wavelength vs. temperature was constructed for each FBG, and linear regression was used to extract an average temperature sensitivity of 0.010 nm/°C for the FBG sensors. Before fatigue tests, three thermocouples were fastened to the surface of specimens, such that each one was located just above one of the FBG sensors in order to monitor the temperature increase due to autogenous heating in the vicinity of the corresponding FBG sensor. Then, the surface temperature data of each thermocouple were converted into the wavelength shift using the previously-determined temperature sensitivity coefficient, and the wavelength changes due to the increase in temperature were subtracted from the corresponding FBG wavelength data at each data point. Here, it should be noted that the surface temperature may not be the same as that sensed by the embedded FBG; however, it provides temperature information very close to the exact values, thereby allowing for higher accuracy in the measured strain.

**Figure 2:**
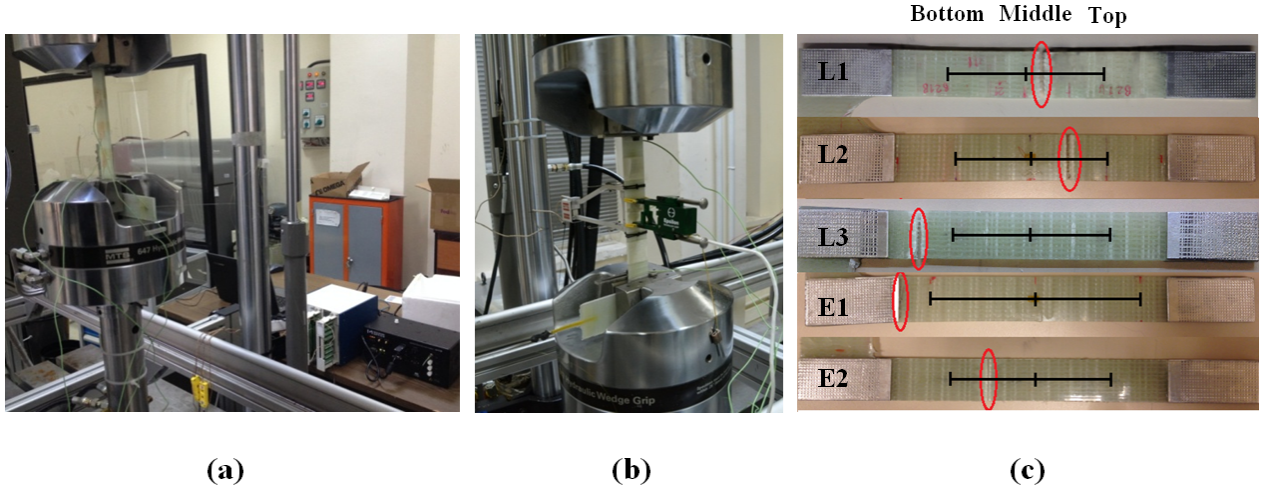
(**a**) Fatigue testing system with the data acquisition set-up; (**b**) the specimen E2with double axial extensometers; (**c**) failed specimens where failure locations are marked with the red circles and sensor locations are indicated by black vertical ticks.

The behaviour of FBG sensors under different experimental conditions was also investigated by utilizing two different experimental procedures, namely fatigue experiments controlled by the LVDT and the extensometer, as tabulated in Table 1. Three of the five presented fatigue experiments (i.e., L1, L2 and L3) were performed under LVDT control, whereas the remaining two experiments (E1 and E2) were conducted under the extensometer control. For experiments with LVDT control, the specimen of interest was subjected to a constant displacement corresponding to the desired strain ratio through using the LVDT sensor of the fatigue testing system. It is recalled that the displacement recorded by the LVDT was related to the strain acquired by the extensometer through a calibration coefficient as described previously. Prior to fatigue experiments controlled by the LVDT sensor, the extensometer was dismounted from the specimens, and the fatigue experiments were performed between minimum and maximum displacements imposed by LVDT. As for the experiments with extensometer control, the extensometer was mounted onto the specimens and kept thereon throughout all of the fatigue tests. The specimens were strained between the minimum and maximum strains corresponding to desired strain ratios. After fatigue tests, the relevant sections of the broken fatigue specimens (Figure 2c) were used to cut out cross-section samples using a circular saw blade for microscope examination of the microscopic details of the failed samples, particularly the interfaces between FBG and host composite material. Their pertinent surfaces were polished using different grits of abrasive papers and inspected under optical microscope and a Leo Supra 35VP Field Emission Scanning Electron Microscope (SEM).

## 3. Results and Discussion

**Figure 3:**
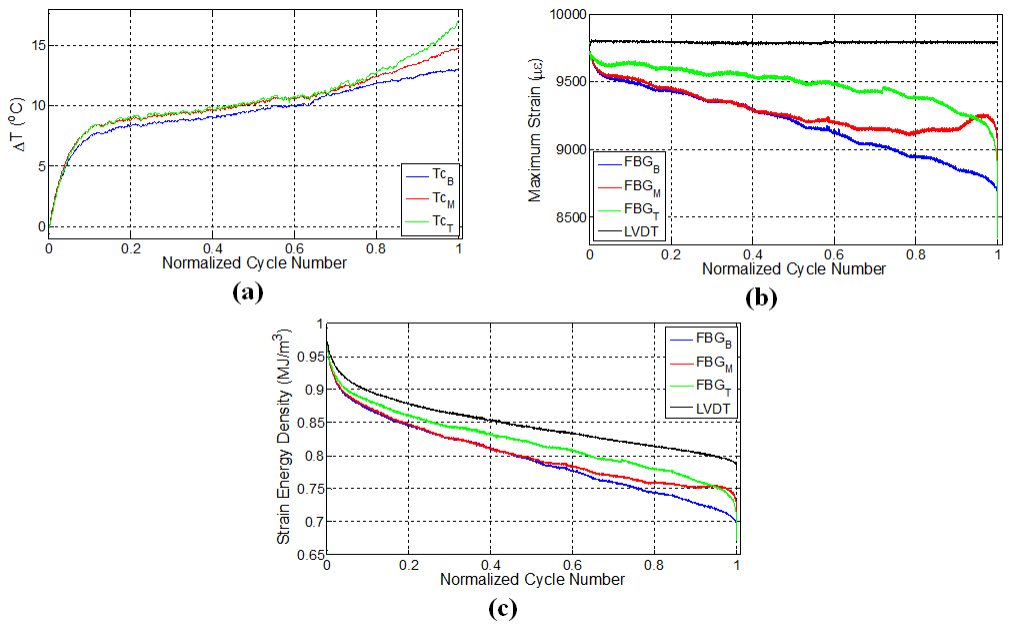
Evolution of (**a**) temperature; (**b**) strain and (**c**) strain energy density for specimen L1 where temperature is monitored by thermocouples, while the strain data are obtained using both FBG and LVDT sensors. The letters B, M and T in subscripts refer to the sensor locations: bottom, middle and top, respectively.

**Figure 4:**
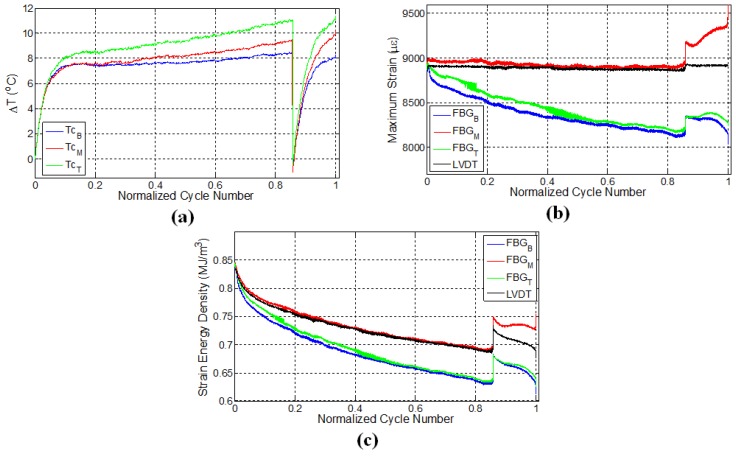
Evolution of (**a**) temperature; (**b**) strain and (**c**) strain energy density for specimen L2.

**Figure 5:**
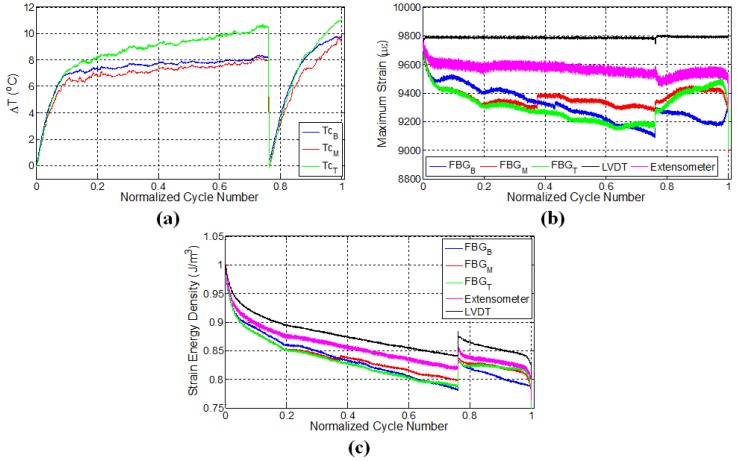
Evolution of (**a**) temperature; (**b**) strain and (**c**) strain energy density for specimen L3.

**Figure 6:**
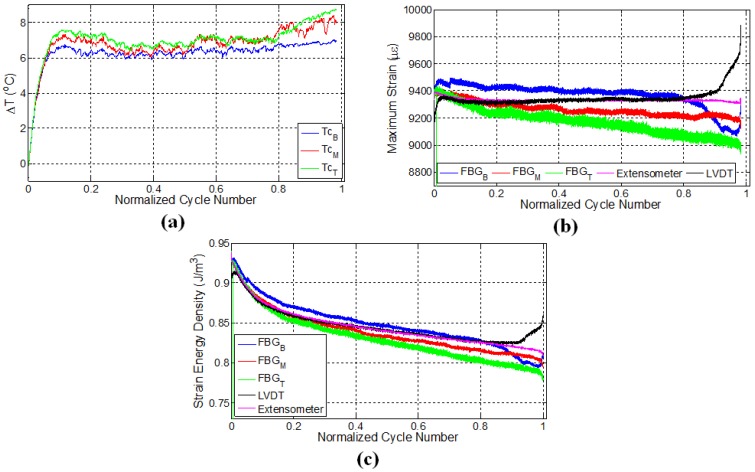
Evolution of (**a**) temperature; (**b**) strain and (**c**) strain energy density for specimen E1.

**Figure 7:**
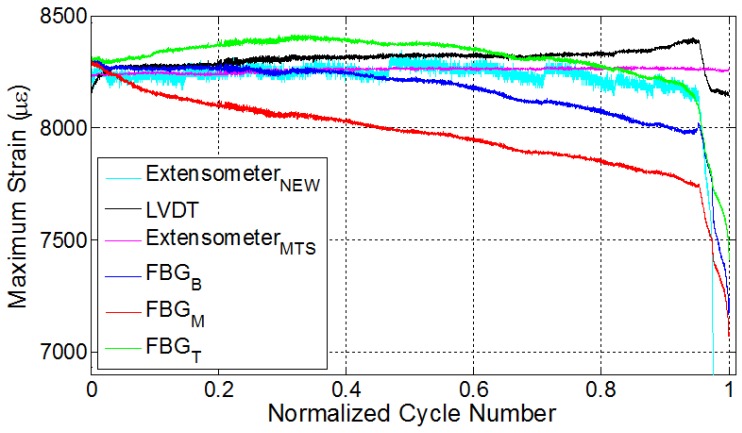
Evolution of strain for specimen E2.

Data acquired from relevant sensors throughout the fatigue tests of specimens were processed and presented as temperature, maximum strains at each cycle and strain energy density variations as a function of cycle numbers, as shown in Figure 3, Figure 4, Figure 5, Figure 6 and Figure 7. The global strain energy density *F* in the specimen was calculated, using F=0.5σε where σ=S/A with *S* being the force measured by the testing machine, A the cross-sectional area of the specimen normal to the force and *ε* the longitudinal strain, either measured using external gages (global) or local FBGs. Here, it should be noted that for all specimens given in Table 1, the intervals between the subsequent FBG sensors are 4 cm, except for the specimen E1, for which it is 6 cm, as shown in Figure 2c.

### 3.1. Specimen L1

Figure 3a shows the variation of surface temperatures for the specimen L1 at three different FBG sensor locations, which were recorded using a K-type thermocouple at a sampling rate of 100 Hz. As can be observed, the temperature evolution reveals three distinct stages: an initial increase called Thermal Stage I, followed by a second phase (Thermal Stage II) for which the rate of temperature variation (in this case temperature increase) is smaller than Thermal Stage I, and finally, a notable increase prior to failure, which is identified by Thermal Stage III. There could be several main contributing factors to the autogenous heating as stated previously, namely viscoelastic/damping effects, plasticity and frictional rubbing of the surfaces of the internal cracks that formed during the test. Intuitively speaking, for specimen L1, the temperature is expected to be noticeably higher for the top FBG sensor, which measures visibly larger local strain, since the higher the strain, the larger the local strain energy in the specimen (as seen in Figure 3c); therefore, one would expect more heat dissipation. This type of behaviour reveals that the heat generated at locations where the FBG sensor reads low strain is not of viscoelastic or plastic origin. If one assumes a fully viscoelastically behaving specimen, the total strain energy accumulated in the volume $F_{\Sigma }=0.5\int_{V}\sigma \varepsilon dV$ is fully converted to heat upon removal of the force on the system under adiabatic conditions using the first law of thermodynamics $dU=F_{\Sigma }$ where dU is the internal energy to be converted to heat after one fatigue cycle. The adiabatic condition assumption is the most conservative one that indicates the heat generated after N number of cycles will remain in the specimen that can be written as $\Sigma_{N}dU=C_{p}\Delta T$ stating that after *N* cycles, the temperature of the specimen will increase by ΔT in proportion to the average heat capacity, $C_{p}$. We take $C_{p}$ as a linear combination in proportion to the volume fractions of the constituents $C_{p}=\alpha C_{p}^{Fibre}+\left(1-\alpha \right)C_{p}^{Matrix}$ where *α* is the volume fraction of the fibres, and $C_{p}^{Fibre}$ and $C_{p}^{Matrix}$ are the heat capacities of the fibres and the matrix at constant pressure, respectively. Thus, one can find the final temperature after N cycles as $\Delta T=\frac{1}{2C_{p}}\sum_{N}\int_{V}\sigma \varepsilon dV$. For $C_{p}^{Fibre}=0.810$ J/g/°C and $C_{p}^{Matrix}=0.12$ J/g/°C, elastic modulus of the fibres, $Y^{Fibre}$, and the epoxy, $Y^{Epoxy}$, being 100 GPA and 3 GPA, respectively (bearing the same strain), and for our specimen having dimensions 3 mm × 25 mm × 150 mm with *N*≈ 30,000 until failure, as in our experiments, we find that the final temperature of the specimen will differ from the ambient test environment by less than a degree, a significantly lower value than observed in our tests. Moreover, that viscoelastic heating should increase with increased local strains is just the opposite of what we observe in our specimen: temperatures are in general the highest where the strain is the lowest. Henceforth, the contribution of the viscoelasticity to temperature increase in the specimens can be ruled out, leaving us with plasticity effects and rubbing of internal crack surfaces, which form with progressing fatigue.

Eliminating the viscoelastic-induced heating as a possible mechanism that gives rise to the observed sample temperatures and noting that the static tensile tests do not give rise to any detectable heating of the specimen, despite the strain being several times more than that of the fatigue test, it can be concluded that heating due to plasticity effects is also negligible, leaving us with only the crack surface rubbing as the main contributor to autogeneous heating. We again remind here that the highest temperatures are locally reached where strain is relaxed. Indeed, upon inspection of Figure 3, the middle FBG sensor region has almost the same temperature value, possibly differing by a degree or so, compared to the top FBG region despite that strain being visibly higher in the middle FBG region, again helping us rule out that global heating during the test is due to plasticity effects. An identical argument was also put forth in the work of Lang and Manson [45], where what is called here “heating due to surface rubbing” is termed as “frictional heating” in that work.

It has been shown in a previous work [5] that, after the initial fast rises in the temperature (Thermal Stage I), there is a gradual and linear decrease in the temperature, which is followed by a level off behaviour (Thermal Stage II). This decrease in temperature is possibly due to the fact that the rate of heat generation is smaller than the rate of heat given off to the environment (as a combination of conduction, convection and radiation, albeit being small), since that test was done at low strain. Thus, the heat generation rate due to damage accumulation is relatively low. In this work, the almost linear rise in temperature of the specimen at the second stage in Figure 3a is attributed to the fact that the damage sites and new surfaces forming in the course of this fatigue experiment are able to generate heat at a rate higher than the heat removal rate. This implies that the higher the crack damage density in the structure, the higher the heat generation, since the temperature rise occurs even though strain energy input to the specimen drops down in accordance with the decline in force due to the damage evolution in the material. At a later stage, around 75% of the fatigue life (1.6 × $10^{4}$ cycles), there is a noticeable deviation in temperature from the linear region for the top and middle thermocouples, such that the temperature rises are augmented, indicating the onset of Thermal Stage III. It is worth mentioning that the temperature regimes observed in this study correlate quite well with the first, second and third phases of the strain energy density versus cycle number curves for the composite material in Figure 3c. However, it is interesting to see that at the locations where temperature rises towards the end of Stage II and beginning of Stage III, the strain data read from FBGs at these locations are changing significantly, implying that portions of the matrix into which the FBG is embedded have a tendency to “detach” from the load-bearing portions still adhered to the fibres.

Figure 3b shows the evolution of maximum strains (i.e., peak strains in the sinusoidal strain form) as a function of cycle number, which was recorded by LVDT and FBG sensors. Recalling that the fatigue test on this specimen was conducted under constant displacement using the LVDT sensor, one may at first expect that FBG sensors should also give constant strain values. However, maximum strains recorded by FBGs can be significantly different in comparison to the global strain of the specimen. As the fatigue experiment progresses, the local strains measured by FBG sensors drop down such that the trend has three separate regions consisting of an initial sharp decrease superseded by a gradual and almost linear decline followed by a final sharp variation after which failure occurs. The drop in the FBG recorded strain as the fatigue experiment continues is due to the damage formed within the specimen, in turn leading to an elongation in the gage length of the specimen. Hence, it is expected that the fatigue equipment should apply less force to induce the desired maximum displacement, whereby the specimen effectively experiences less local strain. Interestingly, these three stages are in agreement with the fatigue phases observed in temperature and strain energy density (based on LVDT) versus cycle number plots in Figure 3a,c, respectively. Besides, each FBG sensor reads notably different local strains, and the relative difference in the FBG measured strains further increases as the fatigue test continues, thereby demonstrating the clear existence of the non-uniform strain distribution due to the local differences in the damage type, density and evolution along the specimen gage length. Moreover, near failure, the strain of the middle FBG sensor starts to increase, while the top one decreases notably, again a sign of inhomogeneous damage accumulation. Note that the corresponding temperatures for these two sensors’ locations increased drastically in the third thermal stage, as well. Such sudden changes in the strain values may signify the possible formation of major deformation other than fibre-matrix debonding and delamination and can be used as an alert for an approaching catastrophic failure. It is interesting to note that the specimen failed at a location close to the middle FBG between the middle and top FBG sensors. These findings also indicate that discrete embedded FBG sensors are reliable in predicting an approaching failure, which would not have been possible otherwise with externally-attached strain gages, especially at high strain fatigue, as strain gages mounted on the surfaces of the specimen can experience debonding from the specimen surface with progressing loading cycles and lose their performance at earlier stages of fatigue experiments.

Figure 3c presents a plot of the strain energy density versus cycle number for all sensors. One can clearly notice that strain energy density calculated using LVDT-based strain possesses three distinct phases. The sudden drop in the strain energy density towards the end is due to the fibre breakage and is caused by the relaxation of applied force on the specimen. As the strains acquired from FBG and LVDT sensors differ, so do their corresponding strain energy density, and FBG sensors experience a larger decrease in their respective strain energy density compared to the one calculated based on LVDT. The variation of strain as a function of cycle number for FBG sensors in Figure 3b resembles that of strain energy density in terms of having initial sudden change followed by a linear region. The difference in the duration of the first phase detected based on the FBG strain and LVDT-based strain energy density can be associated with the fact that the strain field of the sensor with a larger gage length, such as the extensometer and LVDT, is affected by all of the matrix cracks along the gage length, whereas FBG strains are influenced only by the local cracks in the vicinity of the sensor having a much shorter gage length. Therefore, the first phase demarcated based on the FBG strain is slightly shorter. Please note that the strain energy curves given here are obtained by taking the product of the global stress and local strain. Considering the fact that the local strains are read from the FBGs embedded inside the epoxy matrix and that we cannot measure local stress, one should use $F=0.5Y^{Epoxy}\varepsilon^{2}$ where $Y^{Epoxy}$ is the Young’s modulus of the matrix is and *ε* is the local strain. Therefore, the differences in the strain energy curves computed based on $F=0.5Y^{Epoxy}\varepsilon^{2}$ will be more considerable than what has been given in Figure 3c. Going back to the viscoelastic heating, if one uses the relation $\Delta T=\frac{1}{2C_{p}^{Epoxy}}\sum_{N}\int_{V}Y^{Epoxy}\varepsilon^{2}dV$, which stands for local heating in a local epoxy volume under adiabatic conditions, one will find the local ΔT, supposing that the local temperature rise occurs only in proportion with the heat capacity of the epoxy only (that later on dissipates in accordance with the local heat transfer coefficients), still to be much smaller than what is observed globally, proving that viscoelastic heating effects are indeed negligible. In another conservative scenario where the high strain values read by the local FBG are representative of the fibre strain (due to the assumption that the epoxy is perfectly adhered to a local fibre and carries the same strain as that of the fibre), and one modifies the above relation such that $C_{p}^{Epoxy}$ and $Y^{Epoxy}$ are replaced by that of the fibre; ΔT is calculated as 1 °C at most, approaching the values calculated from the global stress and strain values obtained from $\Delta T=\frac{1}{2C_{p}}\sum_{N}\int_{V}\sigma \varepsilon dV$, meaning that fibre viscoelasticity is also not the cause of the temperature.

### 3.2. Specimen L2

A second experiment was performed on the specimen L2 under displacement control with the strain ratio of 0.55 and was designed to consolidate the repeatability of the previous experiment. In this experiment, after 25,000 cycles (80% of the fatigue life), the test was paused; the specimen was kept unloaded for 30 min; and then, the fatigue test was reinitiated while keeping the experimental conditions the same. The specimen failed close to the middle FBG sensor denoted by the red curve in Figure 4b where the local strain has reached a maximum just before the failure. Figure 4a presents the variation of surface temperatures at three different thermocouple locations for specimen L2. For the first fatigue loading, similar to the previous case (Figure 3a), temperatures of all three locations increase sharply and then follow a gradual linear increase, which corresponds to the second fatigue stage. Upon terminating the loading, all temperature values start to decrease as expected. A rather important observation that deserves special consideration is the temperature trend for secondary fatigue loading. Upon re-initiating the fatigue loading, it was noted that the rate of change of temperature was higher than that corresponding to the initial fatigue loading. Moreover, the temperature curve after the pausing-restarting action very rapidly catches up with the curve before the pause, confirming the extensive damage, hence “new surface density” presence in the sample compared to its virgin state. For the latter case, since the specimen is expected to possess much higher damage and related crack density throughout the specimen, friction between the newly-formed crack or damage surfaces act as sources for heat generation in the specimen, thereby increasing the temperature faster compared to the beginning of the test, just like the experiments on the previously-discussed L1 sample.

FBG strains and strain energy densities for the specimen L2 are respectively given in Figure 4b,c. Similar to the results of the previous experiment, FBG strains have a decreasing trend throughout the initial fatigue loading. When the fatigue loading was reinitiated, maximum strains measured by FBG sensors experienced a sudden jump compared to the maximum strain of the last cycle belonging to the first fatigue loading, even though the applied maximum displacement was kept the same. This is due to the thermal strain. It is seen that the specimen has a higher temperature at the end of the first fatigue loading than at the beginning of the second fatigue loading, which implies that a certain portion of the applied strain in the former case is contributed by the thermal strains associated with the thermal expansion of the specimen. When the specimens cool down and the predefined displacement is applied again for the second fatigue loading, the contribution from the thermal strain diminishes, and more force is required to induce the desired displacement onto the specimen, resulting in an upward jump in the measured force. This in turn influences the FBG strains along the specimen, causing it to experience a sudden increase.

After the jump in the strain, the maximum strains start to drop down again until about 28,000 cycles (90% of the fatigue life) for all of the sensors. The rate of these decreases in strain is significantly different compared to that corresponding to the end of the first fatigue loading for all of the respective sensors. At this stage, the strains recorded by top and bottom FBG sensors continue to decrease, whereas the strain of the middle FBG starts to increase, pointing to significant deformations in the vicinity of the middle FBG sensor (recall that a similar behaviour was also noted for the middle FBG sensor of specimen L1). After the restart of the fatigue test, specimen L2 withstands an additional 6000 cycles of fatigue loading before the failure, leading to total of 30,966 cycles to failure. Both L1 and L2 specimens failed at a location close to the middle FBG in the upper part of the specimen (section above the middle FBG), as shown in Figure 2c. The positions at which the specimens have failed are consistent with the abrupt variations in the strain fields presented in both Figure 3b and Figure 4b. Moreover, the failure locations of L1 and L2 specimens coincide with the vicinity of the pair of FBG sensors recording higher local strains on average over the duration of the experiment.

### 3.3. Specimen L3

For the sake of completeness and to validate the possible mechanisms effective in the second experiment, another specimen L3 was prepared and tested. In this test, fatigue loading was applied using LVDT as the control sensor, and an extensometer with the wavelength of 50 mm was mounted onto the specimen to measure the strain during the fatigue loading, such that the centres of the gage length of the extensometer and the composite specimen are aligned. Similar to the specimen L2, two stage fatigue loadings were applied onto the specimen. Initially, fatigue loading corresponding to the strain ratio of 0.6 was introduced for 15,000 cycles (76% of the fatigue life) and after around 30 min; the second fatigue loading was applied with the same maximum displacement until failure, resulting in an additional 4694 cycles to failure. Figure 5 shows the variation of temperatures, strains measured by different sensor systems and the corresponding calculated strain energy densities.

Temperature variations of the specimens showed similar behaviour as in the case of specimen L2 possessing distinct thermal regimes corresponding to the fatigue stages, namely initial rapid increase followed by a gradual increase at a smaller rate, implying that similar damage mechanisms are occurring inside L3, as well. In comparison to the bottom and middle thermocouples, the top thermocouple measures a significant temperature rise in the second thermal stage until the termination of the load, which can be attributed to the higher degree of deformations and related heat generation due to the friction between the surfaces of micro cracks or damage acting as an additional heat source within the specimen. Consistent with the results of specimen L2, the rate of temperature rise during the second loading was higher compared to the first fatigue loading, as the friction between the crack surfaces causes heating within the stress concentration regions already formed during the first loading, generating additional heat during the second loading.

Following the analysis of the strain variations for respective sensors, one can see that the application of constant displacement onto the specimen also causes a decline in strain measured by the extensometer, which is around 300 με. Such a reduction possibly emanates from the non-uniform elongation in the gage length of the specimen, causing less strain transfer to the region falling into the gage length of the extensometer in response to the same imposed global displacement. Another important observation is that the decline in maximum FBG strains is higher than what is read from the extensometer strains. This is likely due to the damage formed in the surrounding of the FBG sensors, which can reduce the effective strain transfer between the local matrix housing the FBG and fibres, thereby causing FBG sensors to read less strain. The reason behind larger drops in the strain of FBG sensors with respect to the extensometer strain can also be related to the difference in the gage lengths of the extensometer (50 mm) and FBG sensors (1 mm), as FBG sensors measure local strains. Again, consistent with the results of preceding experiments, strains measured by different FBG sensors showed different strain quantities due to the non-uniform elongations and damage formation along the specimen. Similar to the previous experiment, the sudden jump in the strain upon the application of the second fatigue loading is caused by thermal strain. At the second fatigue loading, FBG strains behaved rather differently compared to the initial loading, and especially close to failure, there are significant variations in the measured strains pointing to the subsequent incoming catastrophic failure. The maximum strains of bottom FBG sensor significantly increased at the last 1000 cycles (5%) of the fatigue loading, signifying the occurrence of serious damage in the vicinity of the sensor. The specimen failed at a location about 2 cm above the lower grip and about 1.5 cm away from the bottom FBG sensor (Figure 2c). It is important to note that the failure occurred towards the locations of two FBG sensors with the highest strains throughout the experiment. Figure 5c illustrates the variation of strain energy density again calculated via taking the product of the strain read from a given sensor and stress applied by the test machine. There is a good synchronization among the regimes of the temperature, FBG strain and strain energy density variations, keeping in mind that forces applied to the specimen at each cycle vary considerably as the experiments are carried out at constant global strain.

### 3.4. Specimens E1 and E2

Two additional experiments were conducted under constant strain, but this time using an extensometer mounted on the specimens until the failure, where the pertinent specimens are denoted with E1 and E2. Similar to the results obtained from experiments with LVDT control, the decrease in the maximum strain values is also noted for both E1 and E2, wherein FBG strains experience distinctive phases during the fatigue loading process.

Experimental results for the specimen E1 are provided in Figure 6. For this specimen, the imposed strain ratio for the fatigue loading was 0.55. Specific to this specimen, the interval between the subsequent FBG sensors is 6 cm. The specimen failed from the grip location close to the bottom FBG sensor, as can be inferred from Figure 6b, such that the failure is expected to occur in the region near the FBG sensor pair with the largest strain values. Figure 6a shows the local temperature variations along the specimen. Here, the temperatures of all three locations increase sharply due to the autogenous heating, then gradually and slowly drop and then level off since the rate of heat generation is balanced by the rate of heat transfer to the environment, and heat generation related to deformation/damage is not high enough to increase the temperature. The presence of internal damage will alter the effective thermal conductivity of the composite specimen since the discontinuities at cracks act as insulating media. Expectedly, in specimens with larger damage density, the heat generation rate due to damage accumulation or crack formation would be higher than the heat removal rate by the ambient environment in accordance with the k∂T/∂n=hΔT, where $\Delta T=(T_{b}-T_{\infty })$ and $T_{b}$ and $T_{\infty }$ are temperatures of specimen’s surface and the ambient environment; *k* is the thermal conductivity; *h* is the heat transfer coefficient; and ∂/∂n is the spatial derivative along the normal direction. As one can immediately conclude, the smaller the crack density, the smaller the heat generation and the larger the thermal conductivity and the conductive heat flux to the boundary that can be removed from the surface through convection. Therefore, in this specimen, the crack density is envisaged to be smaller than those in the three previous test specimens. This conclusion can be further substantiated referring to smaller deviation of FBG read strains from the strain of the control sensor with respect to former specimens, noting that the higher the damage density, the larger the relaxation of the local strain. Moreover, the maximum temperature attained at the end of the first thermal phase is smaller than those in the former experiments. Finally, after around the 16,000th cycle (83% of the total number of cycles), temperature starts to increase again due to the rate of increase of surfaces via crack growth and related heat generation. Temperature variations for specimen E1 have a rather good correlation with the three fatigue phases, especially for the top thermocouple.

Strain variations for the extensometer, LVDT and FBG sensors are provided in Figure 6b. Recall that using the previously-determined calibration coefficient, the LVDT-recorded displacement was converted into strain. As seen from Figure 6b, until nearly 80% of the fatigue life, the strain values of both extensometer and the LVDT are nearly the same, while FBG sensors have rather different strain values, which are attributed to strain relaxation due to the damage in the vicinity of FBG sensors. After this, the strain of LVDT starts to increase, implying significant deformation or elongation in the specimen to impose the desired strain onto the gage length region of the extensometer (50 mm). In parallel, the strains of the bottom and middle FBG sensors also indicate some notable variations at this stage. Particularly, the strain of the bottom FBG sensor begins to experience a sharper decline with respect to the other FBG sensors, which coincides with the onset of the sudden increase in the LVDT strain. We think that this is related to the formation of major damages and elongations in the vicinity of the bottom FBG sensor signalling the initiation of the third fatigue phase followed by catastrophic failure, which is again consistent with the failure location that occurred at the lower grip from the tab section. A similar behaviour was noted for the bottom FBG sensor of the specimen L3 where failure took place in the similar section of the specimen. Being consistent with all previous cases, the location of the failure is in the vicinity of a region of a pair of FBGs with higher strains throughout the fatigue experiment. Such an outcome is entirely consistent with the scenario that the strain relaxed regions have already failed locally and lost their ability to contribute to the “load-bearing” action and that the rest of the volume with increased strain carries the load, and specimen fracture will occur at these regions. Note that no such conclusion can be drawn from the data of the external extensometer data. For this experiment, the recorded strain trends of the FBG sensors in Figure 6b follow the trend of the strain energy density variation calculated based on the constant strain recorded by the extensometer shown in Figure 6c. Unlike LVDT-controlled experiments, the deviation in FBG strains with respect to the extensometer and LVDT strains is smaller, which is due to the fact that the gage length of the extensometer is much smaller than the gage length of the specimen that LVDT takes into account. Hence, the specimen exhibits a much more uniform strain field, leading to smaller deviation in FBG strains with respect to the imposed/intended global strain. Despite this, the common feature of this specimen with the others before failure is that one of FBG strain signals starts to deviate from the others prominently, in addition to an observable rise in local temperatures.

One should keep in mind that the specimen E2 was manufactured by using the VI method and tested under extensometer control with the strain ratio of 0.5. Unlike the RTM manufactured specimens, this specimen failed at a lower cycle number, which might be attributed to the one-sided rough surface due to presence of peel ply, known to be the source of crack initiation points, or unnoticed possible defects, which are likely formed during the manufacturing. However, the failure cycle of the specimen is irrelevant within the focus of this study. In this test, in order to have further information on the strain variations along the specimen, a second extensometer with a smaller gage length (10 mm) was mounted onto the middle section of the specimen between the two ends of the first extensometer, which inputs the strain data into fatigue testing machine, as shown in Figure 2b. The results of this experiment are presented in Figure 7. The specimen failed at a location between the bottom and top FBG sensors, as shown in Figure 2c. The strain of the second extensometer follows the strain of the first extensometer until ten thousand cycles and, thereafter, deviates from the constant strain value of the first extensometer, highlighting the importance of the gage length and the local dependence of the strain measurement in the specimen experiencing non-uniform deformation. In similitude with previous results, FBG-recorded strains showed a descent over the length of the fatigue experiment. For this experiment, the third phase is quite distinctive in comparison to previous experiments, such that there are abrupt changes and a considerable decline in all FBG-measured strains, as well as in the strain recorded by the second extensometer. It is actually interesting to note that L1, L2, L3 and E1 exhibited a behaviour where at least one FBG would indicate a local increase in strain, concurrent with a local stress increase, whereas no such behaviour took place in this sample. While it is a possibility that the fracture might have started from a location far from any FBG, the “abrupt local strain variations”, namely the third stage before fracture is common, although different in character, with other experiments.

### 3.5. Microscopic Analysis

**Figure 8:**
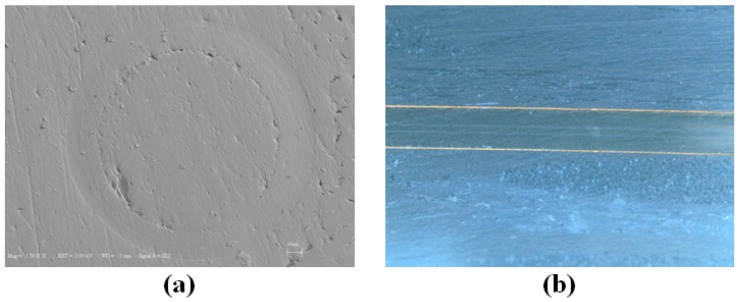
(**a**) Perpendicular and (**b**) longitudinal cross-sections of optical fibres around FBG regions.

During the analysis of the strains acquired by FBG sensors, LVDT and extensometers, we observed that, under constant amplitude, high strain fatigue loadings, the maximum local strains sensed by 1 mm-long FBG sensors can significantly be different from global strain values measured over a larger gage length, and nearly in all experiments, FBG-recorded strains decline. In addition, FBG sensors at different locations have different strains due to non-uniform strain fields or deformation states in discrete regions of specimens. At first sight, one may argue that this decline might be associated with debonding of the sensing part of FBGs and the deterioration of the FBG sensors. The integrity of FBG sensors and their bonding with the host material is very crucial: to check whether debonding of the sensors might have been the case for sudden strain relaxation in some of the FBGs in the experiments, we used SEM and optical microscopy to examine the cross-sections of composite specimens (Figure 8a) and optical microscopy (Figure 8b). No noticeable debonding was detected, as can be seen in Figure 8, which presents the cross-sections taken from the specimen L2, and similar results were obtained for the other failed specimens.

The above observation points out the fact that local strain variations are due to matrix cracking, fibre-matrix debonding and fibre breakages in the vicinity of the FBG sensors, not related to the debonding of FBGs from the matrix. Such cracks, as they accumulate, probably contribute to the observed strain relaxations around the sensor regions, thereby affecting the strain sensed by the sensor. This is not a negative outcome; in fact, the progressive damage modes in the fatigue experiments are well captured by the FBG sensors, such that the variation of strains recorded by FBG sensors can follow the three fatigue phases of the composite corresponding to different internal damage densities. It is again inferred here that it is not possible to observe this effect via the data of the externally-mounted strain gages. In light of all of our experimental findings, it is inferred that the strain relaxation in the vicinity of FBG sensors is one of the contributing reasons for the observed reduction in the maximum strains measured by FBG sensors and is a novel way of predicting an approaching failure. In one of our previous studies [5], FBG-embedded composite specimens with the same constituent materials and stacking configurations as in the case of this study were subjected to a constant strain high cycle fatigue loading (i.e., with the strain ratio of 0.27). It was observed that the maximum strains of the FBG sensors did not experience a severe decline compared to the results of the current work, which suggests that the damage was more limited.

In such experiments, another crucial point that requires special attention is the gage length of the dynamic extensometer used for the fatigue testing can influence the evaluation of the results and, therefore, the damage mechanism dramatically. An extensometer measures the strains within its gage length, i.e., the region between its pins and fatigue testing system impose constant strains only along this region. As a result, the remaining parts of the 150-cm gage length towards the grip sections can show very distinct strain behaviour compared to the middle part of the specimen. It should also be noted that the FBG sensors located at the centre of the gage length experience a decrease in the strain even though they are located within the extensometer gage length range for which the global strain is set to be constant. Thus, local and global strain values can differ significantly from each other, and FBG sensors directly probe the local strain that is reduced or enhanced by the formation of defects. Keeping in mind that strain gradients will reach a maximum near a crack, local readings obtained by FBGs are much more precise and reliable in terms of monitoring the health of the composite and predicting remaining useful lifetime.

At the initial fatigue stage, a sharp rise in the temperature of the composite (first thermal stage) is observed followed by a gradual transition to a linear curve (second thermal stage). The nature of the temperature variation in the second thermal stage differed among the specimens presented in this work. In some specimens (i.e., E1 and E2), the temperature showed a very subtle and gradual decrease followed by levelling off, while in some others (i.e., L1, L2, L3), the temperature was rising at a smaller rate than the one for the first thermal stage. This difference in results can be attributed to the fact that in the latter case, the deformation level was higher and, correspondingly, the rate of heat generation was higher than the rate of heat given off to the surrounding. This effect is also reflected in the strain behaviour of the specimens, such that specimens showing a significant temperature rise in the second thermal stage tend to have higher reductions in the FBG-measured strains due to the stress relaxations caused by the higher degree of deformations. One may thus infer that those specimens showing a temperature rise are likely to have more cracks/deformations than the others. The third thermal stage was generally associated with the sharp rise in temperature, closer to the failure of the specimen despite a decrease in applied load to sustain constant strain.

## 4. Conclusions

Composite specimens containing three sequential FBG sensors embedded along their gage length were exposed to constant, high strain fatigue loads. Considerable differences among the strains recorded by FBG, LVDT and extensometer sensors were observed as the fatigue loading continued, demonstrating that the local and global behaviour of composite materials can be dramatically different. It was found that composite specimens exposed to the same global strain did not necessarily give rise to the same magnitude of strains at the local level. It was demonstrated that such a response from FBG sensors comes from the heterogeneous nature of composites, which leads to a non-uniform strain distribution. While this might not come as a surprise, the interesting strain relaxation behaviour in the vicinity of FBG sensors close to failure via the formation of various damage modes such as matrix cracking and fibre-matrix debonding appears to be related to local heating of the specimen, which was also monitored. Contrary to what one might expect, the highest temperatures were measured at the locations where the lowest strains (or strain relaxation) were recorded, implying that frictional rubbing of surfaces around cracks led to such an outcome. It has also been shown unambiguously that the temperature variations in response to fatigue loading show three different stages, which contribute to damage-induced heat generation. Upon application of the second fatigue loading as in the case of specimens L2 and L3, the temperature rises to the same temperature levels in a much shorter time during the second loading compared to the first fatigue loading, directly revealing the contribution of the friction between the crack surfaces. One important conclusion that is drawn from the study is that the significant deviations in local strains and a noticeable increase in temperature of the composite regardless of location can be used as a signal of an oncoming catastrophic failure. Sensor gage length is also shown to be a crucial factor to consider in such experiments, as constant strains imposed using an extensometer do not necessarily induce similar strains over other strain sensors.

## Figures and Tables

**Table 1:** Test parameters for fatigue experiments. LVDT, linear variable differential transformer.

Specimen Code	Strain Ratio	Fatigue Method	Fatigue Life (Cycle)
L1	0.60	LVDT	20,951
L2	0.55	LVDT	30,966
L3	0.60	LVDT	19,701
E1	0.57	Extensometer	19,385
E2	0.50	Extensometer	13,811
